# Oxidation of Erythrocytes Enhance the Production of Reactive Species in the Presence of Artemisinins

**DOI:** 10.3390/ijms21134799

**Published:** 2020-07-07

**Authors:** Ioannis Tsamesidis, Pierre Pério, Antonella Pantaleo, Karine Reybier

**Affiliations:** 1 Pharma-Dev UMR 152, Université de Toulouse, IRD, UPS, 31000 Toulouse, France; pierre.perio@univ-tlse3.fr (P.P.); karine.reybier-vuattoux@univ-tlse3.fr (K.R.); 2 Department of Biomedical Sciences, University of Sassari, 07100 Sassari, Italy; apantaleo@uniss.it

**Keywords:** artemisinin, phenylhydrazine, oxidized erythrocytes, superoxide radicals, hydrogen peroxide, LC-MS

## Abstract

In red blood cells, hemoglobin iron represents the most plausible candidate to catalyze artemisinin activation but the limited reactivity of iron bound to hemoglobin does not play in favor for its direct involvement. Denatured hemoglobin appears a more likely candidate for artemisinin redox activation because it is expected to contain reactive iron and it has been described to release free heme and/or iron in erythrocyte. The aim of our study is to investigate, using three different methods: fluorescence, electron paramagnetic resonance and liquid chromatography coupled to mass spectrometry, how increasing the level of accessible iron into the red blood cells can enhance the reactive oxygen species (ROS) production derived from artemisinin. The over-increase of iron was achieved using phenylhydrazine, a strong oxidant that causes oxidative stress within erythrocytes, resulting in oxidation of oxyhemoglobin and leading to the formation of methemoglobin, which is subsequently converted into irreversible hemichromes (iron (III) compounds). Our findings confirmed, using the iron III chelator, desferrioxamine, the indirect participation of iron (III) compounds in the activation process of artemisinins. Furthermore, in strong reducing conditions, the activation of artemisinin and the consequent production of ROS was enhanced. In conclusion, we demonstrate, through the measurement of intra-erythrocytic superoxide and hydrogen peroxide production using various methods, that artemisinin activation can be drastically enhanced by pre-oxidation of erythrocytes.

## 1. Introduction

Dioxygen and iron constitute two of the major components of human erythrocytes and for this reason red blood cells (RBCs) have the potential to catalyze the production of highly toxic reactive oxygen species (ROS). Most of the major mutations in human erythrocytes linked to an iron overload and the subsequent production of ROS are hemoglobinopathies, such as thalassemia [1] and sickle cells disease (SCD) [2,3]. Iron commonly mediates ROS production, where iron cycles back and forth between +2 and +3 states and in the process generates ^•^OH free radical via the Fenton reaction [4]. In the erythrocyte, where ^•^OH free radical will lead to Hb denaturation and further release of heme iron, this process can be autocatalytic, leading an ever increasing oxidative stress once it is initiated by the release of threshold amounts of free iron [4,5]. Not surprisingly, healthy RBCs are equipped with multiple mechanisms to inactivate potent oxidants (superoxide dismutase, catalase, glutathione peroxidase, peroxiredoxins, glutathione, methemoglobin (metHb) reductase, etc.), thereby suppressing this auto-catalytic expansion of free iron, allowing them to circulate for 120 days before oxidative stress begins to promote their demise. In the case of hemoglobinopathies, antioxidant compounds were reported as a therapeutic strategy [6,7,8].

On the contrary, one of the most potent drugs involving the production of ROS mediated by iron is artemisinin (ART) and its derivatives, which contain an endoperoxide moiety that can be activated by iron to form cytotoxic reactive species. This characteristic was applied for many pathologies including malaria [9], cancer [10] and osteoporosis [11].

Malaria parasites experience a special challenge with oxidative stress when they invade a human RBC and attempt to use the amino acids in hemoglobin (Hb) for proliferation of their progeny [12]. As Hb is consumed, free heme is released. The solution evolved by the parasite has been to polymerize released heme into a polymer termed hemozoin that is largely inactive in catalyzing ROS production [13]. The selectivity of artemisinins (ARTs) (the most potent antimalarial drugs currently in the clinic) for parasitized erythrocytes derives from their requirement of free iron to mediate their activation [14], whereas the abundance of accessible ferrous iron in healthy RBCs is too low to mediate opening of artemisinin’s endoperoxide ring, suggesting that this process occurs rapidly in parasitized cells where the iron content is much higher [15,16,17]. In addition, it should be borne in mind that the existence of a free ferrous (Fe^2+^)-heme during hemoglobin degradation and hemozoin formation has never been unequivocally demonstrated [18,19].

In cancer, the therapeutic strategy relies on the fact that cancer cells contain significantly more intracellular free iron than normal cells making them more sensitive to artemisinin [14]. It has been shown that artemisinin and its analogs selectively cause apoptosis in multiple cancer cell lines [10,20,21,22,23,24,25,26]. Moreover, artemisinin loaded with transferrin in liposomes demonstrated anticancer activity [27]. A similar mechanism of action for artemisinin has been observed for diseases associated to osteoporosis. In detail, ARTs revealed osteoprotective effects associated with excessive intracellular production of ROS, which leads to inhibition of osteoclast differentiation (responsible for bone loss) by blocking pathways involved in the receptor activator of nuclear factor kappa-B ligand (RANKL) and finally promoting osteogenesis [11]. Considering the characteristic of high levels of intracellular iron in osteoclasts, ART compounds could inhibit osteoclast differentiation via mechanisms associated with intracellular iron, as assumed for the ARTs treatment in malaria parasites.

There is consensus on the need of reactive iron to generate pharmacologically active artemisinin radical species. In malaria, hemoglobin iron represents the most plausible candidate to catalyze artemisinin activation but the limited reactivity of iron bound to hemoglobin does not play in favor for its direct involvement [28]. Denatured hemoglobin appears a more likely candidate for artemisinin redox activation because it is expected to contain reactive iron and it has been described to release free heme and/or iron in erythrocytes [28]. Hence, one strategy that can be used to increase the level of iron is to oxidize RBC using phenylhydrazine. It is well known that phenylhydrazine (PHZ) causes oxidative stress within erythrocytes resulting in oxidation of oxyhemoglobin leading to the formation of metHb, which is subsequently converted into irreversible hemichromes (HMCs) that lead to the precipitation of denatured hemoglobin in the form of Heinz bodies [29]. Methemoglobin has also been reported as a redox-responsive nanocarrier to trigger the in situ anticancer ability of artemisinin [22]). Li et al. demonstrated that the encapsulation of ART into the metHb nanocarrier activated iron-mediated free radical generation and, consequently, triggered an elevated in situ tumor-reducing capacity, proving the promising anticancer ability of the metHb-ART complex. In this paper, we demonstrate, using three different methods: fluorescence, electron paramagnetic resonance (EPR) and LC-MS, how modifying the redox state of red blood cells can potentiate the production of ROS derived from artemisinin activation.

## 2. Results and Discussion

### 2.1. Global Evaluation of ROS Production for Oxidized RBCs in the Presence of Artemisinins

**Figure 1:**
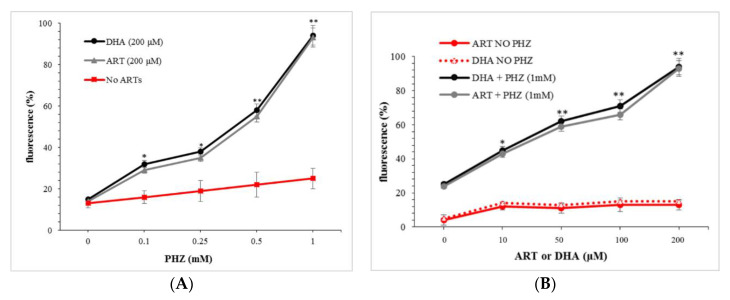
Reactive oxygen species (ROS) levels measured using CM-H_2_DCFDA. (**A**) Red blood cells (RBCs) treated with a fix concentration of artemisinin (ART) and dihydroartemisinin (DHA) (200 μΜ) after pre-treatment with PHZ (0.1–1 mM) and (**B**) RBCs pre-treated with 1 mM PHZ and then ART or DHA (10–200 μΜ). Data are the average ± SD of 5 independent experiments. Significant differences to untreated RBCs at * *p* < 0.05; ** *p* < 0.001.

The oxidation of RBCs was performed using phenylhydrazine (PHZ) as previously reported [30]. PHZ is described as the most potent oxidizing agent as it can oxidize leading to the production of a number of oxidative products [31,32]. The global measurement of ROS produced in the presence of artemisinins was carried out initially using the permeable fluorescent probe CM-H_2_DCFDA. The amount of ROS was first evaluated in the presence of 200 µM artemisinin (ART) or dihydroartemisinin (DHA), the active metabolite of all artemisinin compounds, after pre-treatment (4 h) with increasing concentrations of PHZ. The corresponding results are presented in Figure 1A. The dosage of 200 µM of ARTs was selected in order to be able to discriminate each condition.

Figure 1A shows that the higher the concentration of phenylhydrazine, the higher the production of ROS in the presence of ART or DHA. There were no significant differences between ARTs. Moreover, the production of ROS increased exponentially at a high dose of PHZ. In the absence of ARTs, a linear PHZ dose-relationship was observed too. This phenomenon is in agreement with previous studies which demonstrated that PHZ and its derivatives slowly oxidize to form ROS, the reaction catalyzed by trace transition metal ions and, consequently, induced the formation of oxidized hemoglobin [31,33,34,35].

To confirm this interdependency between the oxidation level of erythrocytes and the amount of artemisinin in the production of ROS, the same set of experiments was carried out for different concentrations of ARTs at the fixed PHZ concentration of 1 mM, identified as the highest concentration to oxidize RBCs without promoting lysis [30,36]. The corresponding results are presented in Figure 1B. These results indicate, as expected, that the amount of ROS originating from the metabolization of ART was drastically increased for PHZ-treated RBCs compared to the untreated ones. Moreover, in the absence of the PHZ treatment, the level of ROS was very weak and did not increase with ARTs, indicating the need of hemoglobin byproducts in the activation of artemisinin derivatives. Indeed, oxidation of hemoglobin produces metHb that is followed by oxidative denaturation and conformational distortions to form hemichromes and heme [37,38,39,40,41]. The same phenomenon is described in pro-oxidant mutations, such as SCD, where accelerated denaturation of Hemoglobin S hemichrome formation and release of heme may collectively induce oxidative stress within the RBC. Antioxidant compounds have been produced in order to prevent oxidative stress presenting antisickling effects [7,8,42].

This conversion generates accessible iron for the activation of artemisinin via the endoperoxide bridge and the subsequent production of ROS [43]. In these conditions, the production of ROS originating from artemisinin is enhanced when increasing the iron pool available into the RBCs as it is the case in malaria-infected erythrocytes where the degradation of hemoglobin-releasing heme takes place [44,45]. In light of these results, to undoubtedly demonstrate the implication of the RBC oxidation byproducts in the artemisinin activation, the ROS produced were specifically detected and quantified using LC-MS.

### 2.2. Specific Evaluation of Superoxide Radicals and Hydrogen Peroxide in Oxidized RBCs in the Presence of Artemisinins

**Figure 2:**
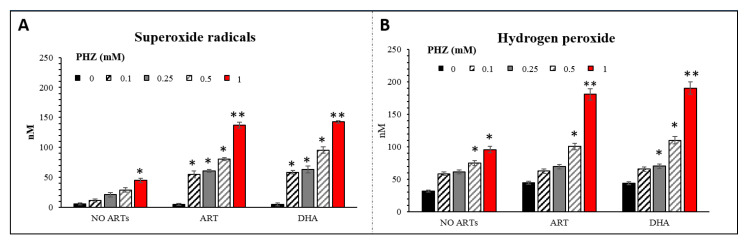
Superoxide radicals (**A**) and hydrogen peroxide (**B**) concentrations deduced from LC-MS analysis in RBCs treated with different concentrations of PHZ (0.2–1 mM) in fix concentration of DHA and ART (200 μΜ). Data are the average ± SD of 5 independent experiments. Significant differences to untreated RBCs at * *p* < 0.05; ** *p* < 0.001.

**Figure 3:**
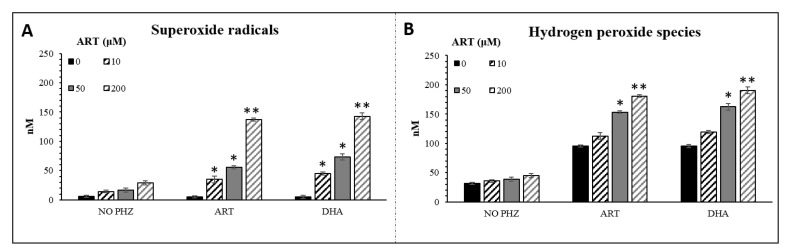
Superoxide radicals (**A**) and hydrogen peroxide (**B**) concentrations deduced from LC-MS analysis in RBCs treated with ARTs (0.2–1 mM) and PHZ (1 mM). Data are the average ± SD of 5 independent experiments. Significant differences to untreated RBCs at * *p* < 0.05; ** *p* < 0.001.

The CM-H_2_DCFDA probe lacks specificity for the targeted reactive species and the autofluorescence of RBCs could influence the interpretation of our data. To better understand and validate our results, a new approach to measure superoxide radicals (O_2_^•^^−^) and hydrogen peroxide (H_2_O_2_) in red blood cells using LC-MS was carried out as previously described [46]. The method is based on the detection of a specific adduct formed into the cell after reaction with DHE and CBA probes for O_2_^•^^−^ and H_2_O_2,_ respectively. The determination of superoxide and hydrogen peroxide are based on the detection of their reaction product 2OH-E^+^ and COH, respectively [47,48]. The O_2_^•^^−^ and H_2_O_2_ levels, measured following the same set of experiments as for CM-H_2_DCFDA, are shown in Figure 2. The concentrations of both ROS were significantly higher in PHZ-treated RBCs compared to the untreated ones (Figure 2). The highest increase was observed in 1 mM-PHZ-treated RBCs in the presence of ARTs. In this case, one can see that the level of hydrogen peroxide is 25% higher than the superoxide one, indicating the probable dismutation of O_2_^•^^−^ into H_2_O_2_. Oxidized RBCs treated with ARTs present a statistically significant PHZ dose-dependent increase of both O_2_^•^^−^ and H_2_O_2_ in comparison with the untreated oxidized RBCs. No significant difference was observed between the tested artemisinins. Both artemisinins induced the production of both reactive species and especially the H_2_O_2_ derived from superoxide dismutation. In addition, the same set of experiments was carried out with different concentrations of ARTs at a fix concentration of PHZ (1 mM) (Figure 3). As expected, the higher the concentration of ARTs, the higher the production of both reactive species indicating a linear ART-dose-relationship for both ARTs. LC-MS analysis confirmed the fluorescence results giving more in depth details about the nature of the ROS produced and their concentration. Moreover, a positive correlation (R = 0.94, *p* < 0.001) between the two methods in parallel was observed indicating the reproducibility between our results. In this study, we decided to investigate the production of superoxide since it is the first radical produced by one electron reduction of oxygen. This radical easily transforms into H_2_O_2_ by dismutation that can be catalyzed by superoxide dismutase. The formed H_2_O_2_ can then either react with transition metal to give the hurtful hydroxyl radical through the Fenton reaction or be transformed into water by catalase enzymes. Few studies have reported that patients with uncomplicated malaria (*P. falciparum* or *P. vivax*) have lower catalase levels and a higher superoxide dismutase (SOD) level than healthy control ones [9,49,50]. An increased SOD activity associated with a reduced catalase activity leads to accumulation of hydrogen peroxide that will rapidly react with accessible ferrous iron to form hydroxyl radicals, promoting damages to essential biomolecules [50]. Moreover, there is no doubt that superoxide is implicated in the mechanism of action of artemisinin since SOD-like compounds was shown to drastically decrease the efficacy of artemisinin on cancer cells [16].

Taken together, all these results confirmed that the activation of artemisinins and the subsequent production of ROS can be enhanced by pre-oxidizing the red blood cells. This enhancement is linked to the formation of denatured products of hemoglobin, may be ferro-protoporphyrin IX, known as responsible of artemisinin activation rather than free ferrous iron [17].

### 2.3. Correlation of Total Reactive Species with Hemoglobin Byproducts

**Figure 4:**
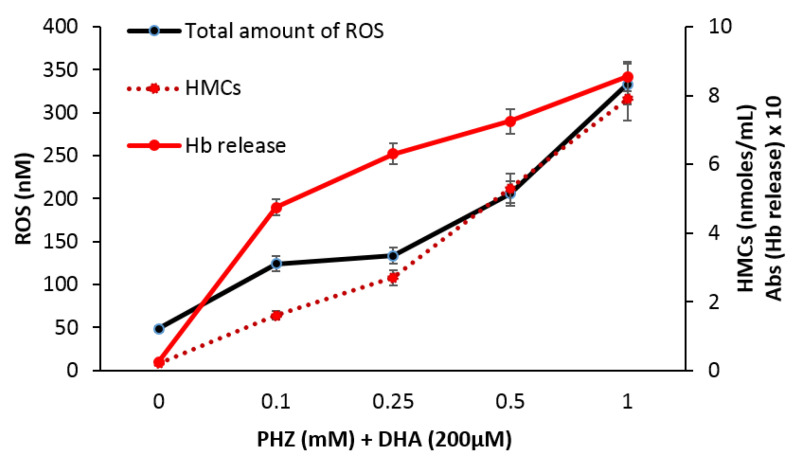
Hemichromes (HMCs) accumulation and total amount of reactive species (O_2_^•^^−^ + H_2_O_2_) in RBCs treated with DHA (200 μΜ) and different concentrations (0.1, 0.25, 0.5, 1 mM) of phenylhydrazine (PHZ).

Ferrous iron is known to be necessary for the activation of artemisinins in parasitized RBCs [17], cancer cells [51,52,53] and osteoclasts [54]. It has been demonstrated that PHZ induces into erythrocytes the efflux of denatured hemoglobin products as hemichromes, heme or even free iron. Figure 4 shows a strong correlation between the total amount of reactive species deduced from Figure 2 and hemichromes accumulation (R = 0.98, *p* < 0.001) as well as other hemoglobin byproduct release (R = 0.87, *p* < 0.05) measured by spectrophotometry at increasing concentrations of PHZ.

**Figure 5:**
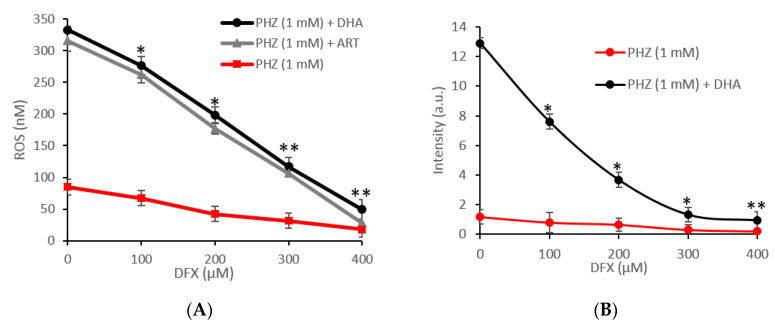
Effect of desferrioxamine (DFX) on the production of ROS measured by (**A**) LC-MS and (**B**) electron paramagnetic resonance (EPR) for RBC treated with phenylhydrazine (PHZ) (1 mM) and DHA (200 µM) or ART (200 µM). Data are the average ± SD of 5 independent experiments. Significant differences to untreated RBCs at * *p* < 0.05; ** *p* < 0.001.

**Figure 6:**
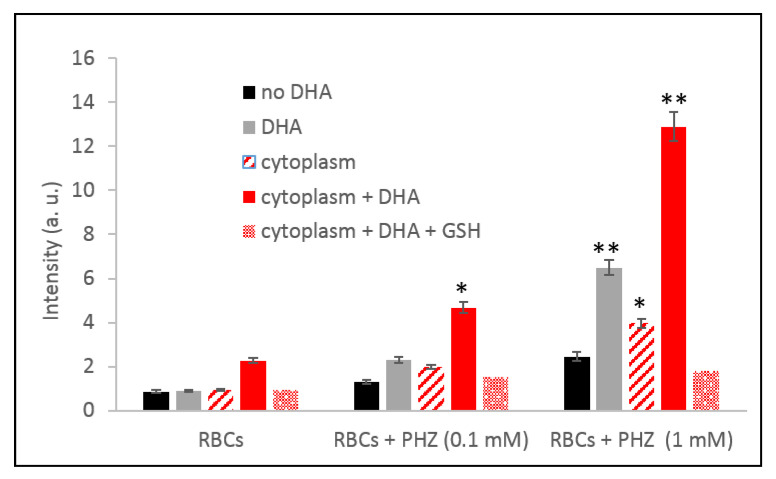
Production of ROS measured by EPR in PHZ-treated and untreated RBC in the presence of their respective cytoplasm enriched with NADPH (nicotinamide adenine dinucleotide phosphate) or GSH 3 mM. Data are the average ± SD of 5 independent experiments. Significant differences to untreated RBCs at * *p* < 0.05; ** *p* < 0.001.

In favor of a causal relationship between hemichromes accumulation and hemoglobin byproduct release and total amount of reactive species, it should be noticed that both phenomena become evident from 0.1 mM concentration of PHZ. These results indicate the possible indirect participation of iron (III) compounds in the activation process of artemisinin. To confirm this hypothesis, the amount of ROS produced was measured in the presence of desferrioxamine, an iron III chelator [55]. In all cases, the activation of artemisinin was inhibited by desferrioxamine as demonstrated in Figure 5 using LC-MS (Figure 5A) and EPR measurements (Figure 5B). These results confirmed the implication of the accumulation of denatured hemoglobin as accessible iron in the activation process of artemisinin to produce radicals. Furthermore, as no radicals were measured by simple incubation of hemichromes with artemisinin, we hypothesized that the reactivity of hemichromes was made possible in reducing conditions. This hypothesis was verified by measuring the ability of the cytoplasm, supposed to contain many reducing metabolites, to induce over-production of radicals in RBCs treated with artemisinin and/or PHZ. The cytoplasm was enriched with reduced nicotinamide adenine dinucleotide phosphate (NADPH) as co-substrate of most redox enzymes. The corresponding results are presented in Figure 6. These results clearly demonstrate that, in strong reducing conditions, the activation of artemisinin and the consequent production of ROS was enhanced. It also confirms the role of Fe(III) byproducts. Moreover, the amount of ROS produced was drastically reduced when replacing NADPH with glutathione (GSH), a strong antioxidant able to trap radicals and oxidative species (Figure 6).

In conclusion, we demonstrate through the measurement of intra-erythrocytic superoxide and hydrogen peroxide production using various methods, that artemisinin activation can be drastically enhanced by pre-oxidation of erythrocytes by phenylhydrazine. This phenomenon is imputable to the consequent accumulation of hemoglobin byproducts as heme or hemichromes that occur in oxidized erythrocytes. Furthermore, these byproducts include iron (III) compounds that can be transformed, in reductive conditions, into the reactive iron (II) form needed for artemisinin activation.

## 3. Materials and Methods

Unless otherwise stated, all materials were obtained from Sigma-Aldrich, St. Louis, MO, USA.

### 3.1. Blood Sample Collection

Whole blood samples from healthy donors, sex-matched with an average age of 45 ± 4.2 years, were collected in EDTA (EthyleneDiamineTetraacetic Acid)-containing tubes in the morning and were centrifuged at 200× *g* for 5 min at 4 °C to separate the cellular components from red blood cells.

### 3.2. Ethics Statement

Healthy donors, all adults, provided written, informed consent before entering the study. The study was conducted in accordance with Good Clinical Practice guidelines and the Declaration of Helsinki. Ethical approval to perform the present study was obtained from the local ethical committee of the ASL 1-Sassari.

### 3.3. Treatment of Red Blood Cells

To stimulate HMC formation, RBCs were suspended at an hematocrit of 30% and incubated with different concentrations (0, 0.1, 0.25, 0.5, 1 mM) of phenylhydrazine (PHZ) at 37 °C for 4 h as previously described [30]. Each reaction was terminated by three washes with phosphate buffer saline containing glucose (PBS-glucose). For all protocols described, untreated controls were processed identically except that the stimulant/inducer was omitted from the incubation.

### 3.4. Fluorescence Assay

For the detection of intracellular reactive oxygen species (ROS), we employed the cell-permeable ROS-sensitive probe 2′,7′-dichlorodihydrofluorescein diacetate (CM-H_2_DCFDA) as previously introduced [56], which fluoresces at 520 nm (λ_ex_ 480 nm) upon oxidation. To conduct an adequate and well-controlled study that would exclude any possibility to register autofluorescence, controls were prepared without CM-H_2_DCFDA but only with its solvent, DMSO. In fact, no or little fluorescence was observed in control RBCs and ART/PHZ-treated RBCs (without incubation with CM-H_2_DCFDA). Oxidation of CM-H_2_DCFDA (prepared as a 0.5 mM stock solution in DMSO) (incubated for 1 h) in RBCs treated with different concentrations of ARTs and PHZ was monitored by measurement of the fluorescence of the desired RBC suspensions (0.2% hematocrit) in 96-well black-walled microplates (Corning^®^, Sigma-Aldrich, Saint Quentin Fallavier, France) using a SAFAS Xenius (Monaco). The relative fluorescence is expressed as “% maximal emission” as determined with the software ”Xenius”, where maximal emission was defined as the fluorescence emission obtained following addition of 3 mM H_2_O_2_.

### 3.5. Liquid Chromatography Coupled to Mass Spectrometry Analysis

Superoxide radicals and hydrogen peroxide in PHZ-treated RBCs, after 1 h of incubation at 37 °C, were analyzed by liquid chromatography coupled with mass spectrometry (LC-MS) as previously described [46,57]. An Ultimate 3000 UHPLC system consisting of a solvent organizer SRD-3600 with degasser, a high pressure binary gradient pump HPG-3400RS, a thermostated autosampler WPS3000TRS, an oven TCC3000SD, an UV-Visible detector DAD3000 (ThermoFisher Scientific, Courtaboeuf, France) and an LTQ-Orbitrap XL ETD mass spectrometer (ThermoFisher Scientific, Courtaboeuf, France) was used. The detection of superoxide radicals was performed with a dihydroethidium (DHE) probe (Sigma-Aldrich, St. Quentin Fallavier, France; Cat. no: 37291) via the detection of 2OH-E^+^ and the detection of H_2_O_2_ using a coumarin boronic acid (CBA) probe (Sigma-Aldrich, St. Quentin Fallavier, France; Cat. n°: SY3397819310) through the detection of COH. Electrospray ionization (ESI) was performed in the positive and negative ion mode for superoxide and hydrogen peroxide, respectively. Quantitative analysis was performed using Xcalibur software and integrating the signal obtained with the corresponding extracted mass (*m*/*z* 330 for 2OH-E^+^ and *m*/*z* 161 for COH) chromatograms. In order to confirm the identity of the detected compounds, the mass spectrometer was used in FTMS (Fourrier Transform Mass spectrometry) mode at a resolution of 15,000 for 2OH-E^+^ and 7500 for COH. For 2OH-E^+^ detection, chromatographic separation was achieved on a Kinetex EVO C18 column, (2.1 × 100 mm, 1.7 μm particle size) (Phenomenex, Le Pecq, France) at a flow rate of 400 μL/min and column temperature set at 50 °C using an aqueous mobile phase containing acetonitrile. For COH detection, chromatographic separation was achieved on a Kinetex C18 column, (2.1 × 100 mm, 1.7 μm particle size) (Phenomenex, Le Pecq, France) at a flow rate of 500 μL/min and column temperature set at 40 °C using an aqueous phase containing formic acid and acetonitrile.

### 3.6. EPR Assay

The detection of free radicals was carried out using *N*-tert-butyl-α-phenylnitrone (PBN) as a spin trap. PBN (1 M stock solution in DMSO) was added to healthy RBCs treated with different concentrations of PHZ and the volume was adjusted with PBS after the addition of dihydroartemisinin (200 µM in DMSO) or their corresponding isolated cytoplasm (diluted 1:10) as previously described [58,59]. The solution was then transferred into a flat quartz cell (FZKI160-5 X 0.3 mm, Magnettech, Berlin, Germany) for EPR analysis. EPR spectra were obtained at room temperature (RT) using the X-band on a Bruker EMX-8/2.7 (9.86 GHz) equipped with a gaussmeter (Bruker, Wissembourg, France) and a high-sensitivity cavity (4119/HS 0205). WINEPR and SIMFONIA software (Bruker, Wissembourg, France) were used for EPR data processing and spectrum simulation. Typical scanning parameters were scan number, 5; scan rate, 1.2 G/s; modulation frequency, 100 kHz; modulation amplitude, 1 G; microwave power, 20 mW; sweep width, 100 G; sweep time, 83.88 s; time constant, 40.96 ms; and magnetic field, 3460–3560 G. The intensity of the EPR signal was calculated by double integration of the EPR signal.

### 3.7. Hemoglobin Release Analysis

Following centrifugation at 1000× *g*, hemoglobin concentration was measured in the tested supernatants as previously described [59].

### 3.8. Hemichromes Analysis

Phenylhydrazine-treated RBCs were incubated for 1 h with ARTs and then washed by cold PBS. Their hypotonic membranes were prepared at 4 °C as previously described. To solubilize the HMCs and to dissociate the cytoskeletal proteins, membranes were treated with 130 mM NaCl, 10 mM Hepes, 1 mM EDTA and 1.5% C12E8 and incubated under stirring (1400 rpm) at 37 °C for 15 min (Eppendorf ThermoMixer^®^C) [51]. To eliminate insoluble aggregates and debris, detergent-treated membranes were centrifuged for 5 min at 20 °C, 15,000× *g*. To isolate the high molecular weight protein aggregate containing HMCs, the supernatant was loaded on a Sepharose CL6B column and chromatographic fractions were screened by spectrophotometry. The fractions characterized by the absorption spectrum of HMCs and lacking absorption peaks of hemoglobin at 280, 434, 520 nm were collected for the quantitative measurement of HMCs and characterization of its components. HMCs were quantified in the high molecular weight fraction by visible spectrophotometry using the following equation [36]:

(1) $$\left[HMCs\right]=-133\times A_{577}-144\times A_{630}+233\times A_{560}$$

with the concentration of hemichromes to be expressed as nmoles/mL of solubilized membranes.

### 3.9. Data Analysis

Data were analyzed using the SPSS version 22.0 statistical package. Descriptive statistics presented as mean ± standard deviation and frequencies presented as percentages. Pearson’s chi-square test or the chi-square test of association was used to discover if there is a relationship between the categorized data, while Fisher’s exact test was used when expected variables were 2% of the total number of variables. Additionally, the independent sample t-test was used to compare between means. In all statistical analysis, the level of significance (*p*-value) was set at α = 0.05.
